# An obstructing endobronchial lipoma simulating COPD

**DOI:** 10.3402/ecrj.v1.25664

**Published:** 2014-09-12

**Authors:** Pradeesh Sivapalan, Magnus Gottlieb, Merete Christensen, Paul Frost Clementsen

**Affiliations:** 1 Department of Internal Medicine, Copenhagen University Hospital, Roskilde, Denmark; 2 Department of Cardiothoracic Surgery, Rigshospitalet, Copenhagen, Denmark; 3 Department of Pulmonary Medicine, Gentofte University Hospital, Hellerup, Denmark

**Keywords:** chronic obstructive pulmonary disease, benign tumors, asthma, surgical resection, chest CT

## Abstract

Endobronchial lipomas are rare benign tumors of the respiratory tract. Bronchial occlusion may cause parenchymal damage and lead to a misdiagnosis of chronic obstructive pulmonary disease or malignancy. Therefore, both accurate diagnosis and radical treatment of endobronchial lipomas are essential. We describe the case of a 61-year-old man with a history of smoking (40 pack years), dyspnea in exertion, and cough during the past 6 months due to an endobronchial lipoma. Chest computed tomographic (CT) scan revealed a circumscribed polypoid lesion partially obstructing the left lower lobe. The endobronchial lipoma was removed by flexible bronchoscopy, and the patient had complete resolution of symptoms following the procedure. Flexible bronchoscopy was normal at the 3-month follow-up. In addition, clinical characteristics, diagnosis, and treatment of endobronchial lipomas are discussed.

Endobronchial lipomas are rare tumors with incidence reported at 0.1–0.5% of all lung tumors (1). They consist of mature adipose tissue, fibrous components, and normal bronchial epithelium (2). The size of the lesions reported varies from <1 cm to >7 cm (3). Computed tomographic (CT) scan is highly specific and sensitive for adipose density in the lesion (4). Flexible bronchoscopy is essential as it identifies the location of lesion and facilitates collection of tissue for histopathology. Endobronchial lipomas can remain clinically silent for many years, and due to their slow growth they are often diagnosed late (5). Clinical manifestation of an endobronchial lipoma can occur as shortness of breath, cough, recurrent pneumonia, atelectasis, and hemoptysis. Bronchial occlusion could lead to a misdiagnosis of chronic obstructive pulmonary disease (COPD) or malignancy (6). Endobronchial lipomas can be removed by surgical or bronchoscopic procedures.

## Case report

A 61-year-old man presented with shortness of breath, cough symptoms during 6 months and atelectasis of the left lower lobe on chest radiograph. Chest radiograph from 2012 was normal. He had a medical history of smoking (40 pack years), COPD, and had been previously operated for acute myocardial infarct and ventricular septum defect. He disconfirmed weight loss, loss of appetite, chest pain, fevers, and night sweats.

**Fig. 1:**
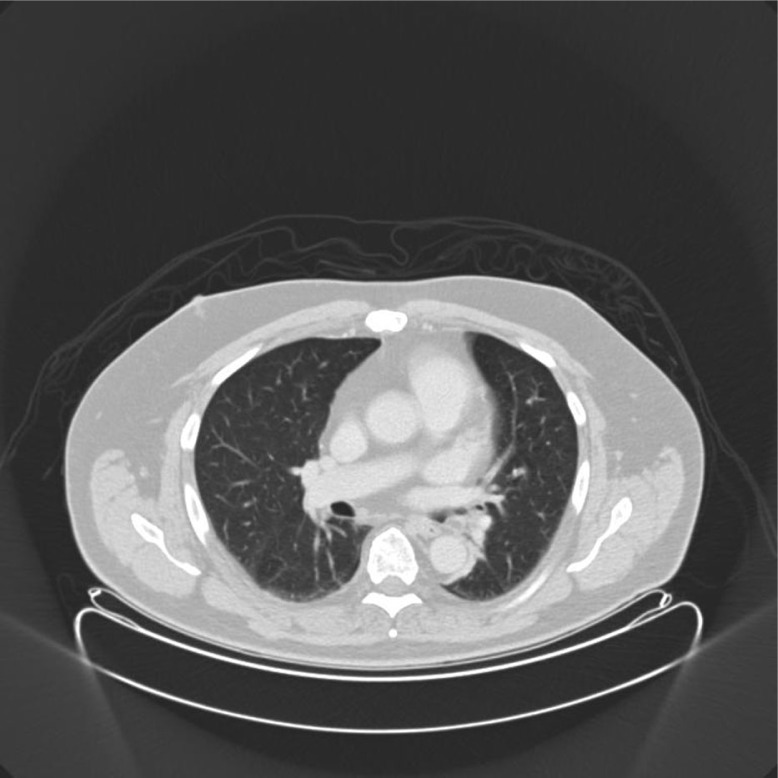
Chest CT before treatment, showing a lesion inside the left bronchial system.

**Fig. 2:**
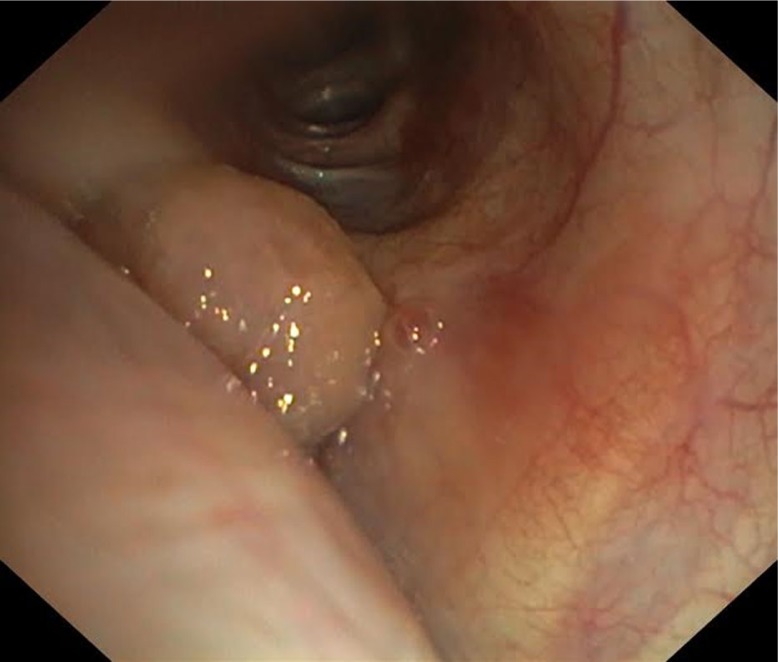
Endobronchial lipoma obstructing segment 6 of the left lower lobe bronchus on bronchoscopy.

Chest CT scan showed the left lower bronchus occupied by fatty content material, size 0.8×0.7×1.1 cm, with atelectasis of the medial segment of the left lower lobe (Fig. 1). Positron emission tomography - computed tomography (PET-CT) did not show any activity in the tumor process. Flexible bronchoscopy examination confirmed the CT findings of a well-circumscribed polypoid lesion partially obstructing the orifice of the left lower lobe bronchus at segment 6 (Fig. 2), and 11 biopsies were performed simultaneously. Biopsy specimens showed only acute inflammatory reactions. Because of indeterminate biopsy specimen, the patient was referred to the department of thoracic surgery for treatment. The lesion was resected using an electrocautery snare followed by argon plasma coagulation at the base of the tumor. Histopathologic evaluation confirmed that the lesion was formed by groups of well-differentiated adipose tissue. In addition, the granulation tissue was seen surrounded by vessel proliferation of lymphocytes, neutrophils, and eosinophils. There was no evidence of malignancy. Thus, the histopathology confirmed endobronchial lipoma as being the diagnosis.

The patient had complete resolution of his symptoms following the procedure. Flexible bronchoscopy was normal at the 3-month follow-up.

## Discussion

Tumors in the tracheobronchial tree are often malignant. Benign lung tumors are rare and comprise 2–5% of all lung tumors (7). Lipomas are common benign neoplasm in soft tissues, though the occurrence of lipoma in the thorax is uncommon. The incidence of endobronchial lipomas among all lung tumors is 0.1% (2). Endobronchial lipomas occur more commonly in males aged 50–70 (8). Smoking and obesity are significant risk factors for endobronchial lipomas (1). In the literature, most endobronchial lipomas originate from the fat cells located in the peribronchial and submucosal tissue of the main or lobular bronchus. Two-thirds of the endobronchial lipomas occur in the right side of the tracheobronchial tree (4, 9). Transbronchial extension is rare. Lipomas in the respiratory tract often occur in the shape of soft, round, or oval mass with smooth surface, poor vascularization, yellowish color, covered with respiratory epithelium (4, 10). The tumors are histologically benign, but a few cases of malignant dysplasia of the surface layer overlying the lipoma have been described (10). Endobronchial lipomas are associated with a significant morbidity and may lead to partial or total bronchial obstruction and secondary lung destruction (11). They are usually misdiagnosed as asthma, COPD, or chronic bronchitis, thus eluding detection for months or years (5). Endobronchial lipomas grow slowly until they obstruct more than 75% of the tracheal lumen before stridor, wheezing, cough, and shortness of breath on exertion occurs (11). Chest radiography is abnormal in 80% of the cases where the typical image is post-obstructive, non-specific changes such as atelectasis, pneumonia, pleura effusions (12), and in time bronchiectasis and empyema (2, 4). However, chest radiography has a low sensitivity (66%) and is not diagnostic for endobronchial lipoma (11). CT scan and magnetic resonance imaging are useful tools in detection of fat-containing lesions, that is, lipoma or lipomatous hamartoma (7). CT scan of the lungs is diagnostic and will demonstrate a fat tissue density without contrast enhancement (8). Flexible bronchoscopic biopsy has a low diagnostic value due to the presence of a solid capsule covering the tumor (7). The differential diagnosis of endobronchial lipoma is mainly to distinguish it from fat-rich pulmonary hamartomas. They may contain of fat, fibrous tissue, bronchial epithelium, and other mesenchymal components (cartilage, bone, smooth muscle, and myxoid tissue) (4, 7).

## Treatment for endobronchial lipoma

Bronchoscopic resection should be considered as the first line of management of endobronchial lipoma. The advantage of bronchoscopic resection of endobronchial lipoma is that it is a less invasive method, preserves lung tissue, resulting in lower morbidity compared with surgical resection. Not only is it diagnostic and therapeutic, it is also associated with good symptomatic control and local control (10, 11).

Both flexible and rigid bronchoscopy have been used successfully (1, 7), although a study by Nassiri et al. concluded that rigid bronchoscopy is superior to flexible bronchoscope for definitive diagnosis (10). The method used for resection (surgical or bronchoscopic) depends on the tumor size, degree of lung damage, the location of the tumor, and the operator's experience (7, 12).

The endoscopic techniques usually include mechanical debulking (13). Additional ablative techniques can be used concomitantly, and they include yttrium aluminum garnet (YAG) laser (10), ethanol injection into the base, electrosurgical snaring (1), argon plasma coagulation of the base (11), and cryotherapy (10). Clinicians should consider surgical procedures (pneumonectomy, lobectomy, and bronchotomy) with possible coexistent malignant tumor, distal irreversible destruction, and in cases of technical difficulties during the bronchoscopic procedure (7).

The recurrence rates of endobronchial lipomas are low after complete surgical resection and the risk of malignant transformation is virtually non-existent (10, 11).

To our knowledge, this unique entity is not previously described in the Danish literature. Clinicians should be aware of this rare condition which may be misinterpreted with symptoms of COPD, asthma, pneumonia, or malignancy. Early diagnosis and intervention should be planned on a case-by-case basis by a multidisciplinary team in centers experienced in complex airway disorders (5, 11).

## Conclusions

The identification and diagnosis of endobronchial lipomas can be challenging as it can simulate obstructive lung diseases. However, early identification and intervention are essential as endobronchial lipomas can cause irreversible damages to the lung parenchyma. Bronchoscopic resection is the first line management of endobronchial lipomas, but surgical resection should be considered as an option in certain complex cases.
